# Tumor microenvironment-induced epigenetic reprogramming of Tregs and its impact on immunotherapy

**DOI:** 10.3389/fgene.2026.1787421

**Published:** 2026-03-27

**Authors:** Wenhao Li, Jiyu Tong

**Affiliations:** 1 Laboratory of Epigenetics and Immunology, West China Institute of Women and Children’s Health, NHC Key Laboratory of Chronobiology, State Key Laboratory of Biotherapy, West China Second University Hospital, Sichuan University, Chengdu, China; 2 Children’s medicine Key Laboratory of Sichuan Province, West China Second University Hospital, Sichuan University, Chengdu, China

**Keywords:** Tregs, tumor macroenvironment, epigenetic, epigenetic regulation, tumor therapy

## Abstract

The tumor microenvironment (TME) represents a complex system comprising various cells and extracellular matrix components that play a crucial role in tumor initiation and progression. While recent therapeutic strategies for predominantly focus on targeting tumor cells, their impact on other cellular components in the TME, such as regulatory T (Treg) cells, remains insufficiently understood. The cellular components of the TME include tumor cells, immune cells, tumor-associated stromal cells, and myeloid-derived suppressor cells. Notably, the role of Treg cells in tumor therapy has emerged as a significant research area of focus in recent years. Regulatory CD4^+^ T cells, characterized by the expression of the transcription factor Forkhead Box P3 (FOXP3) and the surface marker CD25, are pivotal in mediating immune suppression and maintaining immune tolerance and homeostasis. Current tumor treatments mainly rely on radiation and chemotherapy. Although innovative therapies such as immune checkpoint inhibitors (ICIs) and chimeric antigen receptor T-cell (CAR-T) therapies have demonstrated promising outcomes, their efficacy is limited, benefiting only a small subset of patients. Epigenetic inhibitors are increasingly recognized as pivotal in cancer treatment; however, prior research has predominantly concentrated on their effects on the tumor itself, while overlooking the potential influence of these compounds on regulatory T cells (Tregs) within the tumor microenvironment (TME). The therapeutic viability of modulating Tregs within the TME remains uncertain. The intricate microenvironment of the TME significantly influences the distinct epigenetic landscape of tumor-infiltrating Treg cells, including modifications in DNA methylation, histone modifications, and chromatin remodeling. A comprehensive understanding of these epigenetic modifications and the underlying factors driving them could unveil novel strategies for cancer therapy. This approach would enhance the understanding of the critical role of Tregs in tumor therapy and facilitate the development of more effective targeted therapies by addressing the unique epigenetic characteristics of tumor-infiltrating Tregs.

## 1 Introduction

The tumor microenvironment (TME) constitutes a complex ecosystem that plays a critical role in tumor initiation, progression, invasion, and metastasis (Cai et al., 2024). It comprises a diverse array of cells and extracellular components, including tumor cells, immune cells (such as T cells, B cells, macrophages, dendritic cells, etc.), tumor-associated stromal cells (such as cancer-associated fibroblasts, endothelial cells,*etc.*), and myeloid-derived suppressor cells (Zahn, 2017). These cellular constituents engage in intricate signaling pathways and intercellular interactions, collectively shaping the distinctive characteristics of the TME. The TME exhibits multiple functional properties; it provides nutrients and releases growth signals that support the proliferation, invasion, and metastasis of tumor cells. Epithelial cells within the TME secrete chemokine ligand 9 (CCL9) and interleukin-23 (IL-23) to promote angiogenesis and establish an immunosuppressive environment (Kortlever et al., 2017). Furthermore, tumor-associated fibroblasts release transforming growth factor-beta (TGF-β) and platelet-derived growth factor (PDGF), which facilitate tumor cell growth and migration (Qu et al., 2019). Conversely, certain immune cells that infiltrate the TME initiate tumor-killing functions; however, the TME provides numerous mechanisms that enable tumor cells to evade immune detection. Tumor cells can secrete inhibitory cytokines, such as interleukin-10 (IL-10) and prostaglandin E2 (PGE2), which suppress the activity of T cells and natural killer (NK) cells, thereby promoting immune evasion (Liu et al., 2022). Additionally, infiltrating Treg cells (Togashi et al., 2019) and myeloid-derived suppressor cells (MDSCs) (Veglia et al., 2021) within the TME predominantly promote immune evasion by suppressing effector immune cells with anti-tumor functions. Furthermore, tumor-associated fibroblasts release vascular endothelial growth factor A (VEGFA), which promotes tumor angiogenesis (Liu X. et al., 2025). Notably, recent studies have identified a context-dependent subset of “fragile” Tregs that exhibit impaired suppressive stability, characterized by reduced FOXP3 expression or Nrp1 deficiency, These unstable Tregs may acquire effector-like properties, including increased production to tumoricidal cytokines such as INFγ, and thereby contribute to anti-tumor immune responses (Overacre-Delgoffe et al., 2017; Overacre-Delgoffe and Vignali, 2018; Liu X. et al., 2024). The complexity of the TME is a significant contributor to drug resistance and metastasis during cancer treatment, thereby impeding the advancement of cancer therapies (Bejarano et al., 2021). The complexity of the TME is a major contributes to drug resistance and metastasis during cancer treatment, thereby limits the progress of cancer therapies.

Regulatory T cells (Tregs), a specialized subset of CD4^+^ T cells, are integral to the maintenance of immune homeostasis and the suppression of excessive immune responses (Wardell et al., 2025). The Forkhead Box protein P3 (FOXP3) serves as a critical marker for Treg cells and functions as a transcription factor essential for their development and functional maintenance (Josefowicz et al., 2012). FOXP3 is pivotal in mediating the immunosuppressive functions of Tregs, and mutations in the FOXP3 gene are associated with the onset of human Immune Dysregulation, Polyendocrinopathy, Enteropathy, X-linked (IPEX) syndrome, which results in severe autoimmune diseases affecting multiple organs, including the liver, lungs, and skin (Ramsdell and Ziegler, 2014; Ono, 2021). Naturally occurring Tregs (nTregs) are generated in the thymus and inherently possess immunosuppressive capabilities, enabling them to inhibit the activity of both CD4^+^ and CD8^+^ T cells. In contrast, inducible Tregs (iTregs) are differentiated from naïve T cells under specific peripheral conditions. These iTregs are predominantly located in peripheral blood and tissues, where they exert suppressive effects on a broad range of immune cells, including CD8^+^ T cells and NK cells (Li et al., 2020; Savage et al., 2020). Tregs modulate the activation, proliferation, and function of effector T cells through direct cell-to-cell interactions, the secretion of inhibitory cytokines (such as IL-10 and transforming growth factor-bate (TGF-beta)), and the consumption of interleukin-2 (IL-2), thereby preserving immune homeostasis (Nakahashi-Oda et al., 2016; Li et al., 2020). Tregs display a degree of heterogeneity across various tissues, with their function and phenotype adapting to the specific tissue microenvironment. This variability enables Tregs to precisely regulate immune responses under various physiological and pathological conditions (Wyss et al., 2016). Furthermore, Tregs exhibit significant heterogeneity across different tissues, allowing them to adapt to specific microenvironments and precisely regulate immune responses.

Epigenetics refers to the mechanisms that regulate gene expression through DNA and histone modifications without altering the DNA sequence, plays a key role in the development and function of Tregs (Ohkura and Sakaguchi, 2020). Among these epigenetic modifications, DNA methylation is an important mechanism. In Tregs, specific hypomethylated regions, such as the Treg-specific demethylated region (TSDR) of the FOXP3 gene, are essential for maintaining stable Foxp3 expression, thereby ensuring the proper function of Tregs (Morikawa et al., 2014). Histone modifications play a critical role in the regulation of epigenetic processes. For instance, histone H3 lysine 4 trimethylation (H3K4me3) is typically enriched near gene promoters and is frequently associated with the initiation of gene transcription (Wang H. et al., 2023). In contrast, H3K27me3 serves as a key repressive post-translational modification that promotes chromatin condensation and inhibits the recruitment of transcription initiation complexes, thereby suppressing gene transcription (Zhang et al., 2020). In Tregs, these histone modification patterns are intricately linked to the expression of Treg-specific genes, influencing both the development and function of Tregs (Beyer and Huehn, 2017). The targeted deletion of the lysine methyltransferase MML1 in Tregs leads to loss of H3K4me3 at transcriptional start sites, consequently impairing Tregs activation, function, and tissue migration (Wang T. et al., 2024). Additionally, non-coding RNAs, including microRNAs (miRNAs) and long non-coding RNAs (lncRNAs), are implicated in the epigenetic regulation of Treg cells. miRNAs modulated gene expression in Treg cells by binding to target mRNAs, thereby inhibiting their translation or promoting their degradation (Shu et al.). Through various modifications, epigenetics regulates the development, function, and stability of Tregs, thereby maintaining immune balance in the body.

In the TME, Treg cell infiltration is often associated with immune evasion of tumors and poor patient prognosis (Dixon et al.). An increase in Treg infiltration within tumor tissue can suppress anti-tumor immune responses through various mechanisms, thereby promoting tumor proliferation and progression. Treg cells can secrete inhibitory cytokines such as IL-10, TGF-β and interleukin-35 (IL-35), which directly suppress the activity of effector T cells and NK cells within the TME, consequently impairing their tumoricidal functions (Vignali et al., 2008; Wang et al., 2018b). Moreover, Tregs can bind to CD80/CD86 on antigen-presenting cells (APCs) via the cell surface protein cytotoxic T-lymphocyte-associated antigen 4 (CLTA4), thereby inhibiting APC function and suppressing T cell activation (Walker, 2013; Tekguc et al., 2021). Additionally, Treg cells contribute to promoting the immunosuppressive microenvironment by modulating other cellular components within in the TME. For example, Tregs can inhibit the secretion of interferon-gamma (INF-γ) by CD8^+^ T cells, thereby promoting the polarization of macrophages towards the immunosuppressive M2 phenotype (Liu et al., 2019). Additionally, Treg cells can secrete growth factors that promote tumor cell epithelial-to-mesenchymal transition (EMT) and angiogenesis, stimulating the proliferation of fibroblasts and endothelial cells within the TME, thereby enhancing tumor cell proliferation, invasion, and metastasis (Goebel et al., 2015; Li et al., 2020; Ohkura and Sakaguchi, 2020). Finally, Treg cells demonstrate increased metabolic adaptability in the TME, which supports their proliferation and suppressive functions, thereby strengthening the immunosuppressive environment of the TME (Sharma et al., 2024).

Tumor-infiltrating Tregs (TI-Tregs) represent a specialized subset of Treg cells that infiltrate the TME. These TI-Tregs exhibit significant functional and epigenetic distinctions from peripheral Tregs, with epigenetic modifications frequently cited as a pivotal factor underlying these differences (Dixon et al., 2021). Within the TME, TI-Tregs are exposed to various signals that modulate their epigenetic state, ultimately leading to functional changes in these cells. For instance, the DNA methylation patterns of TI-Tregs within the TME are modified, influencing the expression of specific genes (Ohkura and Sakaguchi, 2020). Additionally, chromatin remodeling may enhance the transcription of immunosuppressive genes, thereby enhancing the immunosuppressive capabilities of Tregs. Although Tregs are essential for maintaining immune homeostasis, their presence can impede the efficacy of tumor therapies. A comprehensive understanding of the differences in functional expression, signaling pathways, and surface markers between Tregs in the TME and peripheral Tregs is crucial for the development of targeted strategies aimed at TI-Tregs in cancer treatment. This provides more therapeutic strategies for treating cancer patients.

In this review, we provide a comprehensive overview of the epigenetic characteristics of Treg cells within the TME, including DNA methylation, histone modifications, non-coding RNAs, chromatin remodeling, and lactylation modifications. We further investigate the factors present in the TME that contribute to the epigenetic reprogramming of TI-Treg cells. Furthermore, we review the progress in targeting TI-Treg cells, particularly drugs that specifically target the unique epigenetic features of TI-Tregs. Additionally, based on the unique functional characteristics of Tregs and their significance in tumor treatment, we further discuss the challenges currently faced by strategies targeting TI-Tregs for cancer therapy, and how these issues can be addressed to improve tumor treatment outcomes.

## 2 The TME drives the epigenetic reprogramming of Tregs

### 2.1 The epigenetic features of Tregs in the TME

#### 2.1.1 DNA methylation

DNA methylation is an important epigenetic mechanism that regulates gene expression without altering the underlying DNA sequence, thereby influencing processes such as cell differentiation, development, and disease progression (Jones, 2012; Dai et al., 2021). This process is influenced by variety of upstream signaling pathways, including TGF-β, IL-6, IL-10, which are activated by inflammatory cytokines and other tumor-associated factors in the TME. These signals activate transcription factor like FOXP3 and STAT3, which then initiate specific gene expression changes in Tregs, playing a crucial role in their development and immune-regulatory functions. FOXP3 serves as a pivotal marker for Treg cells, playing a crucial role in their development, differentiation, and function (Fontenot et al., 2003; Hori et al., 2003). The expression of FOXP3 has been observed in various cancers, including pancreatic cancer (Hinz et al., 2007), breast cancer (Liu et al., 2015), and melanoma (Niu et al., 2011), where it exerts different effects (Wang J. et al., 2023; Zhu et al., 2024). Tregs contribute to tumor immune evasion by suppressing the anti-tumor activity of effector T cells through the secretion of inhibitory cytokines, direct interactions with effector cells, and modulation of other cellular components (Vignali et al., 2008; Walker, 2013; Wang et al., 2018b; Liu et al., 2019; Tekguc et al., 2021). Their suppressive function is regulated by epigenetic modifications, particularly DNA methylation (Kumagai et al., 2024). Elevated FOXP3 expression is correlated with its demethylation within the TME (Zhu et al., 2024). In Tregs derived from patients with non-small cell lung cancer, diminished activity of DNA methyltransferases results in the demethylation of eight CpG sites within the FOXP3 promoter. Tumor cells influence the demethylation of the FOXP3 promoter in Tregs, thereby attenuating immune responses and promoting tumor progression (Wang et al., 2011; Zhu et al., 2024). The conserved non-coding sequence 2(CNS2) located within the first intron of the FOXP3 gene encompasses a region rich in CpG dinucleotides, referred to as the Treg-specific demethylation region (TSDR) (Ngalamika et al., 2015), which is crucial for sustaining FOXP3 expression (Baron et al., 2007; Zhuo et al., 2015). In the context of colorectal cancer, STAT5 overexpression in TI-Tregs recruits TET2 to the FOXP3-TSDR, leading to its demethylation and enhancing FOXP3 expression. This interaction between TET2 and FOXP3-TSDR underscores the critical role of epigenetic regulation in Treg function and highlights the complex interplay between different epigenetic layers (Ma et al., 2018). Moreover, increased expression of TET enzymes, coupled with decreased DNA methyltransferase (DNMT) activity, has been shown to result in the hypomethylation of other immune-related genes such as CTLA-4, further enhancing the suppressive function of Tregs in the tumor microenvironment and promoting tumor progression (Sasidharan Nair et al., 2018; Piotrowska et al., 2021). The tumor microenvironment drives demethylation of the FOXP3 and CTLA-4 loci in TI-Tregs. This process is not isolated; rather, it is a coordinated regulation by multiple epigenetic modifications that work in concert with upstream signals and transcriptional networks to fine-tune Treg responses. Further studies are required to elucidate the exact molecular mechanisms and how the TME influences these regulatory networks. Understanding these processes may open new therapeutic avenues for targeting Tregs-mediated immune suppression in cancer therapy.

#### 2.1.2 Histone modification

The development and function of Tregs depend on the stable expression of Foxp3, and the epigenetic regulatory network of Foxp3 involves various histone modifications. Notably, the enrichment of H3K4me3 at the FOXP3 promoter region serves as a critical hallmark of Treg differentiation. This process is influenced by a network of upstream signaling pathways, including TGF-β, IL-6, and IL-10, which activate key transcription factors such as FOXP3 and STAT3. These transcription factors, in turn, regulate the chromatin landscape of Tregs by promoting or inhibiting the binding of various histone-modifying enzymes, further stabilizing FOXP3 expression. Gene mapping analyses of H3K27me3 modifications in Tregs have demonstrated that excessive activation of Enhancer of zeste homolog 2 (EZH2) can initiate H3K27me3 and promote the differentiation of Treg cells into effector Tregs (Peeters et al., 2024). EZH2 is a key histone methyltransferase that mediates H3K27me3 modifications and is essential for maintaining the suppressive function of Tregs under CD28 stimulation (Peeters et al., 2024). The TME provides hypoxic conditions that further enhance EZH2 activity, leading to increased intracellular levels of H3K27me3, which supports Treg differentiation and stability by inhibiting the expression of pro-inflammatory genes. (Zhuo et al., 2015; Ma et al., 2018). Inhibition of EZH2 results in reduced H3K27me3 levels in TI-Tregs and decreases Foxp3 protein expression, consequently diminishing the stability of TI-Tregs. This demethylation process is not isolated; it involves a cascade of histone-modifying events, including the interaction between EZH2 and transcription factors such as STAT5. This cross-talk between histone modification and transcription factor activity is a key regulatory layer in Treg function. EZH2-deficient TI-Tregs secrete pro-inflammatory cytokines such as TNF-α, IFN-γ, and IL-2, while their capacity to produce IL-10 is impaired. This shift results in a pro-inflammatory phenotype that promotes the recruitment of CD8^+^ and CD4^+^ effector T cells to the tumor and enhances their cytotoxic activity (Zhuo et al., 2015; Sasidharan Nair et al., 2018; Piotrowska et al., 2021). Moreover, hypoxia-driven upregulation of EZH2 in the TME facilitates the deposition of H3K27me3 across the genome, leading to the suppression of genes associated with Treg differentiation and function (Goswami et al., 2018). Lysine demethylase 6A (KDM6A) serves as a crucial H3K27me3 demethylase, specifically promoting the demethylation of H3K27me3 (Manna et al., 2015). KDM6A activity is also modulated by upstream cytokine signals, which control its expression in Tregs. Stabilization of KDM6 expression inhibits H3K27me3 demethylation and increases Bcl-2 expression in Tregs, thereby augmenting the anti-apoptotic and suppressive functions of iTregs (Gao et al., 2019). Additionally, the histone demethylase JMJD1 is significantly upregulated in TI-Tregs. As an H3K9me2 demethylase, JMJD1 enhances PD-1 expression while inhibiting AKT and IFN-γ production. Additionally, JMJD1 can directly promote STAT3 demethylation and suppress INF-γ secretion by TI-Tregs (Long et al., 2024). Histone deacetylases (HDACs) and histone acetyltransferases (HATs) function in a reversible manner to regulate the acetylation status of histones H3 and H4 (Dai et al., 2021). In their acetylated state, FOXP3 is stably expressed and exhibits enhanced DNA-binding ability. The knockout of HDAC5 in Tregs reduces the immunosuppressive function of TI-Tregs (Xiao et al., 2016), whereas the knockout of HDAC6 promotes FOXP3 acetylation and strengthens the suppressive function of Tregs (Dahiya et al., 2020). The targeted knockout of HDAC10 in Tregs does not induce autoimmune disease in murine models; however, these cells demonstrated an enhanced suppressive capacity (Dahiya et al., 2020). Conversely, the knockout of HDAC11 results in increased the expression of Foxp3 and TGF-β within Treg cells, thereby enhancing their suppressive function (Huang et al., 2017). The Ep300 gene encodes the adenovirus E1A-associated p300 transcriptional coactivator, which functions as a histone acetyltransferase and modulates transcription through chromatin remodeling (Ghosh et al., 2016; Nicosia et al., 2023). Inhibition of Ep300 leads to a reduction in Foxp3 expression and induces apoptosis in Tregs, consequently impairing their suppressive function and enhancing anti-tumor immunity (Liu et al., 2013). The melanoma antigen family H1 gene (MAGEH1), a member of the type IIMAGE protein family, is expressed across various tissues and operates as an E3 ubiquitin ligase. Comparison of TI-Tregs with peripheral Tregs reveals that TI-Tregs upregulate the expression of MAGEH1 and regulate the ubiquitination status. This process is mediated by upstream signals within the TME, including cytokines and hypoxic factors, and contributes to the survival and suppressive function of Tregs in the tumor microenvironment (Plitas et al., 2016). Histone modifies such as EZH2, JMJD1, HDACs, and p300 modulate Treg stability and effector function through targeted histone methylation and acetylation, thereby enhancing their immunosuppressive activity within the tumor microenvironment and promoting immune evasion.

The distinct epigenetic modifications, including DNA methylation, histone modification, non-coding RNA regulation, chromatin remodeling, and lactylation, contribute to the function divergence between peripheral Tregs and TI-Tregs within the tumor microenvironment.

#### 2.1.3 Non-coding RNA

Non-coding RNAs (ncRNAs), including microRNAs (miRNAs) and long non-coding RNAs(lncRNAs), play critical regulatory roles in Tregs within the TME. In TI-Tregs, the expression profile of ncRNAs is significantly altered, which profoundly impacts Tregs functionality (Ma et al., 2023). MiRNAs regulate gene expression by binding complementarily to target mRNAs, thereby inhibiting their translation or promoting their degradation (McGeary et al., 2019). In addition to direct gene regulation, miRNAs also influence Treg function through modulation of chromatin accessibility and the expression of key transcription factors. For example, miRNAs can modulate the activity of Treg-specific transcription factors like FOXP3 and STAT3, influencing Treg differentiation and immunosuppressive capacity. Previous studies have demonstrated that miRNAs are essential for maintaining the functional stability of Tregs. For instance, miR-21 and miR-155 have been shown to influence the expression of Foxp3 (Stefani and Slack, 2008; Lu et al., 2009), while miR-142-3p regulates GARP expression in Tregs (Zhou et al., 2013) and can indirectly affect Foxp3 levels by targeting AC9 mRNA (Huang et al., 2009; Jebbawi et al., 2014). Notably, the expression levels of certain miRNAs in TI-Tregs differ from those observed in peripheral Tregs, and these differentially expressed miRNAs participate in regulating Treg proliferation, differentiation, and immunosuppressive activity (Xing et al., 2021). Specifically, miR-155 is upregulated in TI-Tregs, where it enhances immunosuppressive functions by targeting key genes such as ICOSL and suppressing effector T cell activity, thereby facilitating tumor immune evasion (Shu et al., 2017; Chao et al., 2019; Tili et al., 2024). In malignant pleural effusions associated with non-small cell lung cancer, Tregs exhibit downregulation of miR-4772-3p, which leads to increased Helios expression and enhances their immunosuppressive capabilities (Yu W.-Q. et al., 2019). Conversely, miR-142-5p inhibits the expression of the cAMP-hydrolyzing enzyme phosphodiesterase, thereby reducing Tregs metabolism and attenuating their suppressive function (Anandagoda et al., 2019). Additionally, miR-125b-5b modulates the immunosuppressive activity of Tregs by targeting TNFR2; its overexpression results in a reduction of both the proportion and suppressive capacity of TI-Tregs, ultimately promoting anti-tumor immunity (Jiang et al., 2022). In the context of hepatocellular carcinoma (HCC), tumor-derived exosomal circGSE1 promotes the proliferation and accumulation of TI-Tregs within the TME through the miR-324-5p/TGFBR1/Smad3 signaling pathway, thereby contributing to tumor progression. This interaction exemplifies how ncRNAs can indirectly regulate Treg function by influencing the TME’s extracellular vesicle-based communication. (Huang et al., 2022). Furthermore, lncRNAs also regulate gene expression through various mechanisms, including interactions with DNA, RNA, or proteins. In breast cancer, lncRNA SNHG1 upregulates indoleamine 2,3-dioxygenase (IDO) and promotes the expression of Foxp3 and IL-10 by inhibiting miR-448, thereby maintaining the immunosuppressive function of Tregs within the TME (Pei et al., 2018). Similarly, lncRNA SNHG16 enhances the TGF-β1/SMAD5 pathway through miR-16-5p, resulting in elevated CD73 expression in TI-Tregs and strengthening their immunosuppressive activity in the TME (Ni et al., 2020). In HCC, the expression of lncRNA FENDRR is reduced, while miR-423-5p is upregulated, which promotes the proliferation of Tregs within the TME and contributes to immune evasion in liver cancer (Yu Z. et al., 2019). These findings demonstrate how ncRNAs integrate with the epigenetic machinery of Tregs to orchestrate immune suppression within the TME, highlighting their potential as therapeutic targets. TI-Tregs exhibit altered expression patterns of non-coding RNAs, including specific microRNAs and lncRNAs. Thes molecular changes modulate Treg proliferation and suppressive function through the regulation of critical genes and signaling pathways, thereby promoting tumor immune evasion and progression.

#### 2.1.4 Chromatin remodeling

Chromatin remodeling refers to the process by which chromatin structure is altered to regulate gene expression. In TI-Tregs within the TME, chromatin remodeling undergoes substantial modifications that significantly influence their functionality (Dixon et al., 2021; Long et al., 2024). Within the TME, the activity of specific chromatin-remodeling complexes is modified, affecting the gene expression profile and functional state of Tregs. Notably, the SWI/SNF family of nucleosome-remodeling complexes plays a pivotal role in regulating Foxp3 expression, with the BAF complex promoting Foxp3 transcription, and the PBAF complex inhibiting it (Loo et al., 2020). In the context of colorectal cancer, alterations in the composition and activity of the SWI/SNF chromatin-remodeling complex in Tregs altered, leading to the upregulation of genes associated with immunosuppression and enhancing the suppressive function of Tregs (Zhao et al., 2020). These alterations are driven by the TME’s inflammatory cytokines, which activate signaling pathways such as the JAK/STAT pathway, and the PI3K-AKT pathway, which, in turn, modulate the activity of chromatin-remodeling complexes and transcription factors. The interaction between these signaling pathways and chromatin-remodeling factors influences Treg differentiation, function, and stability. Single-cell RNA-seq and ATAC-seq analyses of TI-Tregs and peripheral Tregs from non-small cell lung cancer patients have revealed the upregulation of multiple transcription factors, including BATF, NFAT, SOX4, MEF2, and TBX21. This highlights the importance of chromatin-remodeling complexes and transcription factor networks in maintaining the functionality of Tregs under immune stress conditions within the TME. Targeted knockout of BATF in Tregs has been shown to inhibit the activation, infiltration, and chromatin remodeling of TI-Tregs within the TME, ultimately leading to Treg exhaustion (Itahashi et al., 2022). This highlights the importance of chromatin-remodeling complexes and transcription factor networks in maintaining the functionality of Tregs under immune stress conditions within the TME. TI-Tregs require NFAT and AP-1 to enhance the binding of Foxp3 to DNA and regulate the expression of related target genes. The interaction between Foxp3 and chromatin is modulated by antigens and cytokines within the TME, directly influencing gene expression and the functional stability of TI-Tregs (He et al., 2024). Within the TME, functionally altered SWI/SNF chromatin remodeling complexes cooperate with transcription factors including BATF and Foxp3 to reprogram the gene expression landscape of TI-Treg cells, thereby enhancing their immunosuppressive capacity. This complex interplay between chromatin modifications, transcription factors, and cytokine signaling underscores the dynamic regulation of Treg function within the TME.

#### 2.1.5 Lactylation

Tumor cells undergo metabolic reprogramming, converting pyruvate into lactate, which subsequently accumulates within the TME. This accumulation of lactate acts as a signaling molecule, inducing the lactylation of histone lysine residues and stimulating gene transcription (Zhang D. et al., 2019). Specifically, lactate within the TME facilitates lactylation modifications in Tregs (Gu et al., 2022). Lactate-induced histone modifications are tightly regulated by signaling pathways activated within the TME, including the NF-κB and TGF-β pathways, which modulate transcription factor activity and chromatin accessibility in Tregs. Furthermore, lactate induces histone H3K18 lactylation (H3K18la) within the TME, which enhances NF-κb p65 mediated transcriptional activation, upregulates TNFR2 expression, and accelerates the pathological progression of malignant pleural effusion (MPE) (Xue et al., 2024). This process is not limited to direct histone modifications but is also coordinated with other metabolic signals that modulate Treg differentiation and function. In TI-Tregs, lactate-driven histone lactylation increases the transcription of key genes such as CD39, CD73, and CCR8, all of which contribute to the enhanced immunosuppressive capacity of Tregs within the TME (Sun et al., 2023). The increased levels of lactylation in key proteins within TI-Tregs influence their function and the activity of associated signaling pathways. Additionally, lactate in the TME induces the lactylation of Lys72 in MOESIN, which enhances TGF-β signaling pathways and strengthens the interaction between SMAD3 and MOESIN (Gu et al., 2022), thereby promoting the generation of Tregs in the TME and reinforcing the immunosuppressive nature of the tumor microenvironment (Gu et al., 2022). In patients with colorectal cancer, lactate enhances USP39-mediated RNA splicing, thereby promoting CTLA-4 expression in a Foxp3-dependent manner, which enhances the immunosuppressive function of TI-Tregs (Ding et al., 2024). Additionally, lactylation may influence the metabolic state of Treg cells, allowing them to better adapt to the acidic conditions of the TME. For instance, Tregs can increase the expression of the lactate transporter MCT1 in high-lactate environments to maintain their proliferation and suppressive activity (Watson et al., 2021). In summary, the accumulation of lactate within the TME functions as a signaling molecule that enhances the adaptability and suppressive capacity of Tregs by modifying histones and stimulating the transcription of immunoregulatory genes.

Increased glycolysis, lactate accumulation, and nutrient depletion in the tumor microenvironment result in the accumulation of metabolites such as lactate, 2-HG, and adenosine. These metabolites drive epigenetic changes in TI-Tregs, enhancing their suppressive activity. The modifications promote the stability and survival of TI-Tregs, increasing the expression of FOXP3, IL-10, TGF-beta, CD39, CD73, CTLA-4, and PD-1. This strengthens the immunosuppressive environment, facilitating tumor immune evasion.

### 2.2 Epigenetic regulation of Tregs mediated by the TME

**FIGURE 1:**
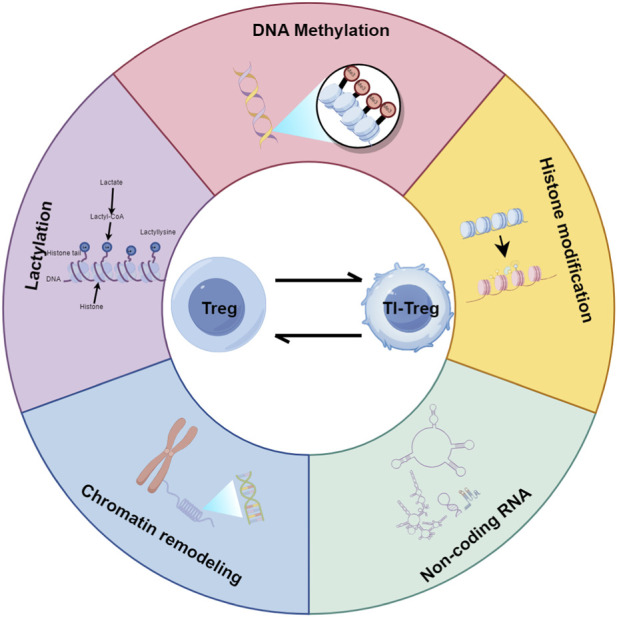
Epigenetic differences between peripheral Tregs and TI-Tregs. This figure was drawn using Figdraw (https://www.figdraw.com).

Various factors within the TME can significantly influence the epigenetic landscape of Tregs, thereby modulating their functional properties and ultimately impacting the antitumor immune response. Epigenetic regulation plays a critical role in controlling Treg cell differentiation and function, involving mechanisms such as DNA methylation, histone acetylation, and chromatin remodeling, which collectively determine the immunosuppressive activity of TI-Tregs (Smiline Girija, 2022). Within the TME, tumor cells secrete metabolites such as TGF-β, lactate, and pyruvate, as well as create hypoxic conditions, all of which can modify the DNA methylation patterns of TI-Tregs (Multhoff and Vaupel, 2021; Xue et al., 2024). Moreover, cytokines such as IL-2, CCL20, IL-17, and IL-6 enhance STAT3 expression in Tregs, thereby facilitating tumor immune evasion (Li and Liu, 2016). Additionally, tumor-associated stromal cells and hypoxia-related signals further modify the epigenetic landscape of TI-Tregs, enhancing their immunosuppressive capacity within the TME (Figure 1) (Macedo-Silva et al., 2019; Sarkar et al., 2022).

#### 2.2.1 Regulation of Treg epigenetics by metabolic metabolites within the TME

**FIGURE 2:**
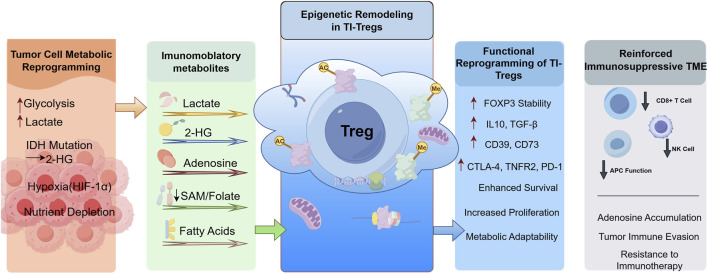
Metabolic reprogramming of tumor-infiltrating Treg cells. This figure was drawn using Figdraw (https://www.figdraw.com).

Metabolic reprogramming of tumor cells within the TME leads to nutrient depletion and the accumulation of immunosuppressive metabolic byproducts. These metabolites have the capacity to modify epigenetic programs and signaling networks, thereby affecting the differentiation, proliferation, and activation of immune effector cells, ultimately modulating the function of Tregs (Li Y. et al., 2021). The metabolite 2-hydroxyglutarate (2-HG), frequently produced by a metabolite commonly produced by IDH mutations in gliomas and acute myeloid leukemia, can alter genome-wide histone and DNA methylation (Xu et al., 2011). The generation of 2-HG directly reduces methylation at the FOXP3 locus, enhancing Foxp3 expression (Xu et al., 2017; Li Y. et al., 2021). In addition, TME-derived metabolites can indirectly regulate Treg epigenetics by influencing their metabolic pathways. Dysregulated glycolysis in tumor cells increases lactate production in the TME (Barbieri et al., 2023), which is closely associated with increased acidity (Brand et al., 2016; Feng et al., 2022). Acidic conditions in the TME disrupt one-carbon metabolism in Tregs, leading to altered intracellular metabolite levels and consequently affecting epigenetic modifications (Mani et al., 2024). Under acidic conditions within the TME, the metabolic reprogramming of nTregs results in alterations to the one-carbon folate pathway, leading to decreased levels of S-adenosylmethionine (SAM), folate, and glutathione. This metabolic adaptation enhances the immunosuppressive activity of nTregs in a sustained manner, thereby promoting tumor immune evasion (Mani et al., 2024). Moreover, the accumulation of lactate in the TME induces histone lactylation in Tregs, which affects the activity of histone-modifying enzymes and alters histone marks (Qu et al., 2023). Lactate functions not only as a metabolic substrate for Tregs but also supports their suppressive function. Tregs modulate their metabolic and functional states in response to nutrient availability. Targeting lactate metabolism directly or mitigating TME acidity can reduce Treg-mediated suppression of cytotoxic immune cells, thereby enhancing antitumor immunity (Watson et al., 2021). Furthermore, hypoxic conditions within the TME upregulate the expression of CD39 and CD73 through hypoxia-inducible factor 1(HIF-1) and TGF-β signaling pathways, resulting in increased ATP hydrolysis and elevated adenosine levels (Antonioli et al., 2013). Adenosine interacts with A2A receptors, influencing intracellular signaling pathways and altering the epigenetic landscape of Tregs, thereby enhancing their immunosuppressive functions (Zhu et al., 2023). TI-Tregs depend on a metabolic network comprising glycolysis, fatty acid synthesis, and fatty acid oxidation to endure and proliferate under challenging conditions (Pacella et al., 2018). These cells acquire and utilize extracellular free fatty acids to fulfill their metabolic requirements, thus supporting their survival and suppressive capabilities through lipid metabolism (Berod et al., 2014; Gerriets et al., 2015; Field et al., 2020). Fatty acid-binding proteins (FABPs), which are integral to lipid uptake and intracellular transport, play a crucial role in this process (Furuhashi and Hotamisligil, 2008). TI-Tregs preserve mitochondrial integrity and regulatory function through elevated expression of FABPs (Rolph et al., 2006). The inhibition of FABP5 in Tregs leads to the release of mitochondrial DNA release and activates the cGAS-STING-dependent type I IFN signaling, resulting in increased IL-10 production and enhanced immunosuppressive activity (Field et al., 2020). Metabolic byproducts and conditions in the TME-including 2-HG, lactate-derived acidity, hypoxia, and adenosine drive epigenetic reprogramming and metabolic adaptation in Tregs via DNA methylation, histone lactylation, disruption of one-carbon metabolism, and FABP dependent lipid metabolism, ultimately bolstering their immunosuppressive function and enabling tumor immune evasion (Figure 2).

#### 2.2.2 The influence of cytokine networks on the epigenetic regulation of Tregs

The cytokine network within the TME plays a crucial role in regulating the epigenetic state of Tregs. Cytokines present in the TME, including interleukins and TGF-β, interact with receptors on Tregs to initiate intracellular signaling cascades that influence the activity of epigenetic enzymes (Yang et al., 2023). Various cytokines affect epigenetic modifications at critical Treg loci through multiple signaling pathways, thereby modulating Treg differentiation, function, and stability (Li and Liu, 2016). In the context of hepatocellular carcinoma, the CCL20–IL-17–IL-6 cytokine signaling axis is instrumental in regulating Treg cell activity. Similarly, in lung cancer, there is a significant increase in CCL20 mRNA expression, which is associated with upregulation of CCR6 on Tregs and elevated levels of STAT3 within these cells. These modifications in the cytokine network promote immune suppression, thereby promoting tumor metastasis (Li and Liu, 2016). Additionally, cytokines can modulate Treg function by influencing the epigenetic modifications of the FOXP3 gene. IL-2 shapes the epigenetic landscape of thymus-derived Tregs by regulating the localization of SATB1, which in turn controls genome-wide chromatin accessibility and Treg functionality (Chorro et al., 2018). TGF-β modulates FOXP3 methylation and the differentiation of Tregs through UHRF1. TGF-β induces Uhrf1 phosphorylation and nuclear exclusion, leading to its proteasome dependent degradation and promoting Treg differentiation (Sun et al., 2019). Furthermore, TGF-β activates Smad signaling and can enhance the expression of DNA methyltransferases (DNMTs), leading to locus-specific modifications in DNA methylation that influence the expression of Treg-relevant genes (Ohkura and Sakaguchi, 2020). Tumor-derived cytokines, notably interleukins and TGF-β, remodel the epigenetic architecture of Treg through signal dependent control of FOXP3 expression and chromatin remodeling, This reprogramming locks in Treg differentiation and stability, constraining antitumor immunity and advancing disease progression.

#### 2.2.3 Tumor-associated stromal cells induce epigenetic reprogramming of TI-Tregs

Tumor-associated stromal cells, including cancer-associated fibroblasts (CAFs) (Liao et al., 2019; Micalet et al., 2024) and tumor-associated macrophages (TAMs) (Zhang R. et al., 2019), constitute critical elements of the TME and significantly contribute to the regulation of the epigenetic landscape of TI-Tregs through cellular interactions (Yang et al., 2023). Within the TME, CAFs secrete a variety of cytokines and chemokines, such as CCL22, CCL18, and CXCL12, which not only recruit Tregs to tumor sites but also impact their epigenetic reprogramming (Wang et al., 2017; Sarkar et al., 2022). CCL22 interacts with the CCR4 receptor on Tregs, initiating intracellular signaling pathways that modify histone modifications and enhance the expression of genes linked to immunosuppressive functions. In patients with breast cancer, FOXP3 and HAT1 in Tregs modify the acetylation status of the CCR4 promoter, thereby enhancing CCR4 expression. These CCR4+ Tregs are subsequently attracted to the tumor tissue by CCL22 and CCL17 secreted within the TME (Sarkar et al., 2022). Additionally, macrophages within TME are activated and polarized towards the immunosuppressive M2 phenotype (Wang S. et al., 2024). Cytokines and metabolites released by these cells attract Tregs to the tumor microenvironment and contribute to their epigenetic remodeling (Wang et al., 2017; DeNardo and Ruffell, 2019; Wang S. et al., 2024). Arginase-2 secreted by M2 macrophages induces alterations in the metabolic state of Tregs within the tumor, leading to modifications in metabolite production and influencing the activity of epigenetic regulatory enzymes. Consequently, the altered metabolic state affects the activity of epigenetic regulatory enzymes, reshaping DNA methylation and histone modification patterns within Treg cells and ultimately enhancing the immunosuppressive capacity of TI-Tregs (Brüne et al., 2015). Tumor-associated stromal cells, including CAF and TAM, actively remodel the epigenetic landscape of TI-Tregs through secreted factors and cellular interactions, such as CCL22-CCR4 signaling and metabolic modulation *via* arginase-2-which enhance the expression of immunosuppressive genes and reshape DNA methylation and histone modification patterns, ultimately strengthening Treg-mediated immune suppression within the TME.

#### 2.2.4 The role of hypoxic condition in the TME in regulating the epigenetic reprogramming of Tregs

Hypoxia is a prevalent characteristic of numerous solid tumors (Godet et al., 2019) and contributes to tumor immune evasion and progression through the induction of epigenetic modifications in Tregs (Macedo-Silva et al., 2019). Hypoxia-inducible factors (HIFs) are activated under hypoxic conditions and play a crucial role in modulating the epigenetic landscape of Tregs, thereby enhancing their immunosuppressive function (Kao et al., 2022; Wu et al., 2022). In the context of esophageal cancer, the formation of hypoxic regions and the activation of HIF-1α are linked to resistance to radiotherapy. Furthermore, hypoxia influences various epigenetic mechanisms, including antigen presentation, cellular stress responses, DNA methylation, and histone methylation, which collectively affect Tregs function within the TME (Macedo-Silva et al., 2019). In addition to direct effects, hypoxia modulates the secretion of tumor-derived exosomes, thereby indirectly regulating the epigenetic state of Tregs. In hepatocellular carcinoma, hypoxia decreases the secretion of miR-101, resulting in macrophage activation and inflammation, which subsequently reshape the epigenetic profile and immunosuppressive capacity of Tregs (Li J. et al., 2021). The hypoxic microenvironment exerts a multifaceted influence on immune cell dynamics, impacting not only the function of Tregs but also modulating the activity of various other immune cell populations. This modulation contributes to tumor immune evasion and progression. In addition to influencing Tregs, hypoxia induces transcriptional activation, polarization, and proliferation of TAMs towards phenotypes that promote angiogenesis and support various regulatory immune subsets, ultimately facilitating tumor growth and metastasis (Kes et al., 2020). Moreover, hypoxia regulates metabolic competition between tumor cells and immune cells, leading to the suppression of effector T cell function and further diminishing antitumor immune responses (Wei et al., 2021). Hypoxia in the TME induced epigenetic remodeling in Treg through HIF-dependent DNA and histone methylation, as well as via indirect routes mediated by tumor-derived exosomes and metabolic competition. These mechanisms potentiate Treg suppressive activity, compromise therapeutic response, and reshapes local immune cell interactions to advance tumor progression.

Various factors within the TME, including altered metabolic profiles, dysregulated cytokine networks, diverse stromal cell populations, and hypoxic conditions, collectively contribute to the epigenetic reprogramming of Tregs. These alterations enhance the immunosuppressive capacity of Tregs within tumors, in part through the upregulation of checkpoint molecules. By increasing the expression of CTLA-4 and PD-1, Tregs significantly suppress the cytotoxicity of effector cells, thereby strengthening tumor-associated immune suppression (Aksoylar and Boussiotis, 2020; Tekguc et al., 2021; Ding et al., 2024). Ubiquitin-specific peptidase 39 (USP39) is a critical component of the RNA splicing machinery that sustains CTLA-4 expression through splicing regulation in Tregs. In the colorectal cancer TME, high lactate levels promote USP39-mediated RNA splicing in a Foxp3-dependent manner, leading to increased CTLA-4 expression (Ding et al., 2024). CTLA-4 is highly expressed on the surface of Treg cells, where it binds to CD80/CD86 on antigen-presenting cells (APCs), competitively inhibiting the interaction between CD28 and CD80/CD86, thereby suppressing T-cell activation and proliferation (Buchan et al., 2018). PD-1 is another critical immune checkpoint molecule. Upon interaction between PD-1 on the surface of Tregs and its ligand PD-L1 on tumor cells or APCs, a cascade of intracellular inhibitory signaling pathways is triggered, leading to the suppression of effector T cell functions (Riley, 2009; Aksoylar and Boussiotis, 2020; Diskin et al., 2020). This suppression not only limits T cell proliferation and cytokine production but also diminishes their cytotoxic activity (Wartewig and Ruland, 2019). In numerous tumors, elevated expression of PD-1 on Tregs enhances their immunosuppressive capacity within the TME, thereby facilitating tumor cells evasion of immune surveillance (Liu Y. et al., 2024). Inhibition of the PD-1/PD-L1 interaction can reinstate T cell functionality and enhance antitumor immune responses (Constantinidou et al., 2019). The clinical application of PD-1 or PD-L1 inhibitors has become a major component of cancer immunotherapy, demonstrating efficacy across various malignancies (Meng et al., 2023), including non-small cell lung cancer (Leung and Van den Eynde, 2022), melanoma (Bhave et al., 2022), and hepatocellular carcinoma (Meng et al., 2023; Guo et al., 2025). The TME integrates metabolic stress, cytokine, stromal signal, and hypoxia to reprogram the epigenetic landscapes of Tregs. The remodeling elevates expression of immune checkpoint molecules, notably CTLA-4 and PD-, through distinct mechanisms-including lactate-driven USP39-mediated RNA splicing that stabilizes CTLA-4 transcripts. Elevated checkpoint expression on TI-Tregs enables competitive sequestration or CD80/CD86, while simultaneously enforcing inhibitory signaling in effector T cell. Thess axis represents a druggable dependency is substantiated by the breadth of clinical activity seen with PD-1/PD-L1 inhibitors.

Treg cells suppress effector cell function and enhance tumor-associated immunosuppression, primarily through the secretion of inhibitory cytokines (Dixon et al., 2021). Tregs are capable of producing a variety of inhibitory cytokines, including IL-10, TGF-β, and IL-35, each of which suppresses effector cell activity through distinct signaling pathways (Sawant et al., 2019). IL-10 inhibits the activity of various immune cells such as macrophages and dendritic cells, thereby reducing their capacity to activate T cells (Mishra et al., 2025). Additionally, IL-10 directly inhibits the proliferation and cytokine secretion of effector T cells, and in the majority of cancer patients, it constrains antitumor immune responses (Chang et al., 2012; Kureshi and Dougan, 2025). The presence of IL-10^+^ Treg cells within tumors is correlated with the prognosis of colorectal cancer patients and serves as a predictor of increased risk for tumor recurrence or metastasis following chemotherapy (Sun et al., 2024). TGF-β exerts more extensive immunosuppressive effects by interacting with myeloid-derived suppressor cells (MDSCs) and regulatory B cells to inhibit the activity of effector T cells and natural killer (NK) cells, thereby promoting tumor immune evasion (Uckun, 2021). It further promotes the differentiation of Tregs, thereby enhancing the immunosuppressive characteristics of the tumor microenvironment. Additionally, TGF-β interacts with receptors on stromal cells, stimulating the synthesis of collagen and extracellular matrix components, which facilitates tumor invasion and metastasis (Dixon et al., 2021; Kureshi and Dougan, 2025). Furthermore, lactate present in the TME can activate TGF-β signaling in Treg cells through the phosphorylation of SMAD3, modulating their metabolic reprogramming and consequently enhancing their suppressive function and adaptability within the TME (Gu et al., 2022). Tregs suppress anti-tumor immunity through cytokines like IL-10 and TGF-β, which inhibits effector cells, promote tumor progression, and enhance their suppressive functions, with lactate in the tumor microenvironment further amplifying TGF-β signaling and metabolic adaptability.

This figure summarizes the key epigenetic targets involved in regulating the suppressive function of Tregs, including DNA methylation-related factors (DNMTs and TET enzymes), chromatin remodeling complexes (SWI/SNF and BRG1), histone-modifying enzymes (EZH2 and HDACs), and non-coding RNA molecules (miRNAs and lncRNAs). These epigenetic mechanisms collectively regulate the stability and suppressive activity of Tregs and represent potential therapeutic targets. Based on these critical targets, multiple intervention strategies have been developed, including EZH2 inhibitors (tazemetostat and CPI-1205), HDAC inhibitors (vorinostat, entinostat, romidepsin, etc.), BET inhibitors (JQ1/OTX015), DNMT inhibitors (decitabine and azacitidine), as well as approaches targeting specific miRNAs/lncRNAs (miR-21, miR-324-5p, SNHG1, etc.). These strategies aim to attenuate Treg-mediated immunosuppression within the TME, thereby enhancing anti-tumor immune responses.

## 3 Therapeutic strategies targeting the epigenetic regulation of Tregs

### 3.1 Epigenetic drugs

**FIGURE 3:**
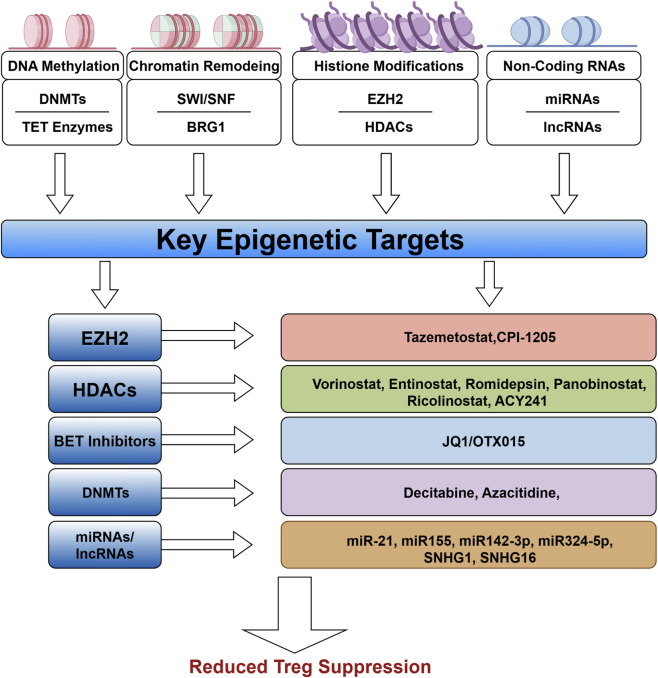
Tumor microenvironment-induced epigenetic reprogramming of Tregs and Its impact on immunotherapy. This figure was drawn using Figdraw (https://www.figdraw.com).

As a novel class of therapeutic agents, epigenetic drugs provide innovative strategies for cancer treatment by modulating the epigenetic states of both tumor and immune cells (Bates, 2020). Currently, several epigenetic drugs have received approval for clinical use, including DNA methyltransferase inhibitors (DNMTis),histone deacetylase inhibitors (HDACis),isocitrate dehydrogenase inhibitors (IDHis), and enhancer of zeste homolog 2 inhibitors (EZH2is) (Dai et al., 2024). DNMTis inhibit the activity of DNA methyltransferases, thereby reducing DNA methylation levels and reactivating genes that are silenced in tumors, ultimately exerting antitumor effects (Lu et al., 2020). For instance, decitabine is a DNMTi approved by the FDA, has demonstrated efficacy in suppressing glioblastoma growth by inhibiting DNA methylation (Lai et al., 2024) (Figure 3). HDACis function by blocking the activity of histone deacetylases, which increases histone acetylation levels, alters chromatin structure, and promotes gene transcription. HDACis such as vorinostat, belinostat, and romidepsin have been approved for the treatment of hematologic malignancies, and their therapeutic potential is being further investigated in other cancer types (Kenny et al., 2020; Horwitz et al., 2024). In the context of cancer immunotherapy, HDACi not only exert direct antitumor effects on cancer cells but also enhance the activation of immune cells such as T cells and NK cells within the TME, thereby improving antitumor responses and patient survival (Pan and Zheng, 2020). The combination therapy involving the HDAC6 inhibitor ricolinostat and the bromodomain inhibitor JQ1 has demonstrated efficacy reducing the immunosuppressive function of Tregs in non-small cell lung cancer, thereby promoting antitumor immunity and effectively prolonging the survival of tumor-bearing mice (Adeegbe et al., 2017). ACY241 is a selective HDAC6 inhibitor has been shown to decrease the number of Tregs in lung cancer and promote the accumulation of TI-Tregs and NK cells within the tumor (Bag et al., 2022). The classIHDACi entinostat enhances the acetylation of STAT3, leading to the downregulation of Foxp3 expression and attenuation of the immunosuppressive function of Tregs, ultimately improving antitumor efficacy (Kang and Zappasodi, 2023). Furthermore, combined treatment with entinostat and IL-2 has been found to further enhance antitumor immune responses in mouse models of renal cell carcinoma (Shen et al., 2012). Similarly, the inhibition of EZH2 through CPI-1205 (Vaswani et al., 2016) has been shown to specifically target TI-Tregs, diminish their immunosuppressive functions, and induce a proinflammatory phenotype. This process contributes to the remodeling of the tumor microenvironment and promoting the recruitment and activation of CD8^+^ and CD4^+^ effector T cells within the tumor (Wang D. et al., 2018). However, the clinical application of epigenetic drugs encounters several challenges, including limited specificity and potential toxic side effects, which require further investigation and optimization (Dai et al., 2024). Epigenetic therapeutics, particularly HDACi and EZH2i hold promise in immunotherapy by reprogramming immune and tumor cell states to enhances antitumor immunity, although challenges related to specificity and toxicity persist (Table 1).

**TABLE 1:** Epigenetic drug targeting Treg cells in cancer therapy.

Drug	Epigenetic target	Mechanism related to Treg/TI-Treg	Cancer type	Key references
Azacitidine (5-AZA)	DNA methylation (DNMT1)	Demethylation of FOXP3 enhancer/promoter	Myelodysplastic syndromes, leukemia	Chiappinelli et al. (2015), Saleh et al. (2015)
Decitabine (5-aza-2′-deoxycytidine)	DNMT inhibitor	Alters Treg differentiation, influences Treg stability *via* DNA methylation changes	AML, melanoma, immune context	Dai et al. (2021)
Vorinostat (SAHA)	HDAC inhibitor	Modulates FOXP3 acetylation, may reduce Treg suppressive function depending on context	Melanoma, breast cancer models	Tian et al. (2025)
Entinostat (MS-275)	HDAC1/3 inhibitor	Downregulates FOXP3/Helios in Treg, reduces Treg suppression; synergize with checkpoint inhibitors	Breast, colon cancer models	Pili et al. (2017), Lin et al. (2021), Kang and Zappasodi (2023)
Romidepsin	HDAC Class I inhibitor	Alters Treg proportions/Th1-Th2/Treg balance in tumor models	T-cell lymphoma, solid tumor immune studies	Kelly-Sell et al. (2012)
Panobinostat	HDAC inhibitor	Reduces TNFR2^+^ Treg in AML; alters immune suppressive phenotype	AML, multiple myeloma immune studies	Govindaraj et al. (2013)
Ricolinostat	HDAC6 inhibitor	Downregulates the immunosuppressive function of Treg cells in NSCLC and enhance anti-tumor response	Non-small cell lung cancer	Adeegbe et al. (2017)
Tazemetostat	EZH2 inhibitor	Destabilises TI-Treg; enhances CD8^+^ T cell responses	Lymphoma, melanoma, solid tumors	Mortezaee (2025)
ACY241	HDAC6 inhibitor	Reduce Treg cells and promote the accumulation of tumor-infiltrating T cells and NK cells within the tumor	Lung tumor	Bag et al. (2022)
CPI-1205	EZH2 inhibitor	Drives TI-Treg to pro-inflammatory phenotype; diminishes FOXP3/Nrp1/Bach2 in Treg	Pre-clinical tumor immune model (anti-CTLA-4 context)	Vaswani et al. (2016), Goswami et al. (2018), Wang et al. (2018a)
JQ1/OTX015	BET family inhibitor	Suppresses FOXP3 and Treg signature genes; reduces Treg frequency; enhances effector T cell activity	Lung, pancreatic, colon cancers	Zhu et al. (2016)
GSK-J4	JMJD3/UTX inhibitor	Inhibits Treg induction by interfering with histone methylation	Glioblastoma, colorectal cancer models	Doñas et al. (2016)
SP-2577 (Seclidemstat)	LSD1 inhibitor	Blocks FOXP3 enhancer demethylation, reduces Treg proliferation; enhances effector T cell activity	Ovarian cancer immune models	Zhao et al. (2025)
JMJD1C inhibitor	JMJD1C histone demethylase inhibitor	Suppresses tumor growth by targeting TI-Treg without affecting systemic Treg	Pre-clinical tumor immune model	Long et al. (2024)

### 3.2 Specific targeting of TI-Tregs with therapeutic drugs

The primary objective of developing drugs that specifically target TI-Tregs is to selectively inhibit or eliminate these cells, thereby enhancing antitumor immunity while minimizing disruption to systemic immune homeostasis (Wen et al., 2025). Current drug development efforts are concentrated on exploiting molecules that are selectively expressed on the surface of TI-Tregs as therapeutic targets. CC chemokine receptor 8 (CCR8) is highly expressed in TI-Tregs but exhibits low expression in normal tissues. Monoclonal antibodies or small molecule inhibitors targeting CCR8 can specifically bind to TI-Tregs and eliminate them either through antibody-dependent cytotoxicity or by interfering with CCR8-related signaling pathways (Kim et al., 2021; Wen et al., 2025). JMJD1C is a histone demethylase that is highly expressed in TI-Tregs and plays a crucial role in maintaining their stability within the TME. Inhibition of JMJD1C selectively targets TI-Tregs, suppressing tumor growth without disrupting systemic immune balance (Long et al., 2024). Furthermore, abnormal activation of certain transcription factors and signaling pathways in TI-Tregs is closely associated with tumor immune evasion, and drugs targeting these molecules are currently under active investigation. B lymphocyte-induced maturation protein 1 (Blimp1) functions as a transcriptional repressor that modulates the balance of effector T cell activity (Nutt et al., 2007). Inhibition of Blimp1 in TI-Tregs can reprogram their transcriptional profile, attenuate their immunosuppressive function, and consequently enhance antitumor immune responses (Dixon et al., 2021). The AAA-ATPase p97 (also known as VCP) participates in multiple cellular processes (Nie et al., 2024), including endoplasmic reticulum-associated degradation (Ye et al., 2001), DNA repair (Krastev et al., 2022), and autophagy (Ju et al., 2009). Npl4 forms a complex with p97 to recognize and extract ubiquitinated protein substrates, facilitating their transport to the proteasome for degradation, thereby maintaining intracellular protein homeostasis. Targeting the p97-Npl4 interaction effectively suppresses TI-Tregs and enhances antitumor immune responses (Nie et al., 2024). The STAT3 inhibitor WP1066 currently in phase I clinical trials, augments antitumor immunity by inhibiting the function of TI-Tregs in melanoma (Kong et al., 2009; Carelock et al., 2023). Therapeutic agents that specifically target TI-Tregs present a more precise strategy for cancer immunotherapy. However, challenges persist regarding their efficacy, safety, and capacity to overcome tumor heterogeneity (Yan et al., 2019). In addition, glucocorticoid-induced tumor necrosis factor receptor (GITR) agonist antibodies, such as DTA-1, have demonstrated the capacity to provoke robust antitumor immune responses across various tumor models. A critical mechanism for enhancing antitumor immunity involves the efficient depletion of TI-Tregs, which substantially inhibits tumor progression (Pan et al., 2022). The administration of anti-KLRG1 antibodies has been shown to selectively deplete Tregs within melanomas without impacting peripheral Tregs. Additionally, combination therapy with JQ1 further enhances TI-Tregs clearance and antitumor activity (Noyes et al., 2022). Efforts to target surface markers, epigenetic regulators, and signaling pathways in TI-Tregs have led to promising preclinical and early clinical candidates. However, achieving selective efficacy without disrupting immune homeostasis remains challenging, with tumor heterogeneity and on-target toxicities presenting significant obstacles (Table 2).

**TABLE 2:** Therapeutic drug targeting TI-Treg in cancer therapy.

Drug	Target/Mechanism	Cancer type	Key references
Denileukin diftitox	IL-2Rα(CD25) IL-2-diphtheria toxin fusion	Various solid tumors; hematologic malignancies	Curiel et al. (2004), Kumar et al. (2019), Thibodeaux et al. (2021), Gwin et al. (2025)
Anti-CD25 antibodies/engineered anti-CD25 mAbs	CD25 (IL-2Rα) – monoclonal antibodies/engineered Fc	Advanced solid tumors	Li et al. (2010), Liu et al. (2011), Son et al. (2015)
Mogamulizumab	CCR4–ADCC-enabled monoclonal antibody	Cutaneous T-cell lymphoma and solid tumor	Ni et al. (2014), Hong et al. (2021)
Anti-CCR8 antibodies	CCR8 – tumor-Treg-enriched chemokine receptor	Multiple solid tumors	Chen et al. (2024), Gampa et al. (2024)
Anti-CTLA-4 antibodies	CTLA-4 checkpoint blockade; Fc-dependent effector functions	Melanoma and other solid tumors	Simpson et al. (2013), Arce Vargas et al. (2018), van Pul et al. (2022)
GITR agonists	GITR-agonist antibodies/ligands	Multiple solid tumors	Davar et al. (2022)
PI3Kδ inhibitors	PI3Kδ–small molecule inhibitor	Hematologic malignancies and solid tumors	Lim et al. (2018)
ADC/immunotoxins targeting Treg markers	Antibody–drug conjugates or immunotoxins delivering cytotoxins to Treg markers	early clinical across tumor types	Yang and Bae (2023)
Metabolic modulators	Metabolic pathways	solid tumor models	Angelin et al. (2017), Mempel and Krappmann (2022)

### 3.3 Combination therapy of epigenetic inhibitors with ICB or CAR-T cell therapy

The integration of epigenetic inhibitors with immune checkpoint blockade (ICB) or chimeric antigen receptor T-cell (CAR-T) therapies demonstrates significant promise in augmenting antitumor immune responses (Liang et al., 2023). ICB therapy counteracts tumor-induced immune suppression by inhibiting immune checkpoint molecules such as PD-1/PD-L1 and CTLA-4, thus activating effector T cells and reinstating their antitumor functionality (Morad et al., 2021). However, a subset of patients exhibits limited responsiveness or develops resistance to ICB therapy. Epigenetic inhibitors have the potential to address this challenge by reprogramming the epigenetic landscape of both tumor and immune cells, thereby enhancing tumor immunogenicity and sensitizing tumors to ICB (Long et al., 2024). DNMTi can reactivate the expression of silenced tumor-associated antigens, thereby increasing tumor immunogenicity across various cancer types. In murine models of ovarian cancer, combination therapy with decitabine and anti-CTLA-4 has been shown to extend the response duration of tumor-infiltrating lymphocytes and significantly enhance therapeutic efficacy (Wang et al., 2015; Liang et al., 2023). HDACi enhance MHC molecule expression and antigen presentation while modulating immune cell activity within the TME to promote immune activation and synergize with ICB therapy (Pan and Zheng, 2020). In prostate cancer, combined administration of the HDACi CN133 with anti-PD-1 therapy has been shown to inhibit the accumulation of TI-Tregs and significantly enhance antitumor efficacy (Chen et al., 2023). Similarly, the EZH2 inhibitor CPI-1205 reduced the suppressive capacity of TI-Tregs while enhancing the cytotoxic activity of tumor-infiltrating effector T cells (Teffs). This effect is further potentiated when combined with anti-CTLA-4 therapy (Goswami et al., 2018). In melanoma models, elevated lactate levels have been observed to enhance the suppressive function of TI-Tregs, whereas the inhibition of lactate dehydrogenase leads to the destabilization of Tregs and improves the effectiveness of anti-PD-1 therapy (Gu et al., 2022). Furthermore, several adenosine A2A receptor (A2AR) antagonists, including ciforadenant (CPI-444), inupadenant (EOS-100850), and AZD-463 are currently undergoing clinical evaluation for cancer treatment (Zhu et al., 2023). Notably, CPI-444 has been found to downregulate multiple immune checkpoint molecules, including PD-1 and LAG-3, on Tregs, thereby enhancing tumor control when used in combination with anti-PD-1 therapy (Ohta et al., 2009; Willingham et al., 2018). In cancer therapy, the integration of epigenetic inhibitors with CAR-T cell treatment has demonstrated potential benefits. These inhibitors modulate the epigenetic landscape of CAR-T cells, thereby enhancing their proliferation and survival, which in turn augments their cytotoxic activity against tumor cells. Additionally, epigenetic modulation can promote the differentiation of CAR-T cells into memory-like subsets, thereby improving their persistence and long-term antitumor efficacy *in vivo* (Funk et al., 2022). Consequently, the combination of epigenetic inhibitors with ICB or CAR-T therapies emerges as a promising strategy to further enhance the efficacy of cancer immunotherapy (Hogg et al., 2020). Combining epigenetic modulators with immune checkpoint blockade or CAR-T cell therapy reshapes the TME by restoring antigen expression, limiting Treg-mediated suppression, and enhancing effector cell function, thereby helping to overcome therapeutic resistance. Preclinical studies support the integration of DNMTi, HDACi, or EZH2i with checkpoint inhibitors, as well as metabolic interventions targeting lactate or adenosine pathways, to improve immunotherapy efficacy and inform clinical translation.

### 3.4 Efficacy and safety of current clinical trials

In recent years, numerous clinical trials have been undertaken to assess the efficacy and safety of therapeutic strategies aimed at modulating Treg epigenetic regulation. Certain patients have exhibited specific therapeutic responses to epigenetic drugs in clinical setting. In hematologic malignancies, combination therapy involving DNMTi and conventional chemotherapeutic agents has been associated with prolonged progression-free survival and enhanced overall response rates compared to chemotherapy alone (Lu et al., 2020). However, these agents frequently induce hematologic toxicity and adverse reactions, necessitating vigilant monitoring and management during treatment. Clinical trials focusing on drugs specifically targeting TI-Treg are also advancing. Monoclonal antibodies directed against molecules such as CCR8 have shown promising safety profiles and therapeutic efficacy in early-phase clinical trials. These antibodies effectively decrease the population of tumor-infiltrating Treg cells while exerting minimal effects on systemic immune homeostasis (Wen et al., 2025). However, larger-scale clinical studies are required to further substantiate their efficacy and long-term safety. In clinical trials investigating the combination of epigenetic inhibitors with ICB or CAR-T therapy, preliminary findings suggest that these combinations can enhance antitumor immune responses and improve therapeutic outcomes in certain patients. However, these combined modalities may also elevate the risk of immune-related adverse events, including pneumonitis and colitis (Liang et al., 2023). Collectively, current clinical trials have yielded significant evidence supporting therapeutic strategies that target the epigenetic regulation of Tregs. Further optimization of treatment regimens is essential to enhance therapeutic efficacy while mitigating adverse effects.

Immunoregulatory functions, complicating efforts to selectively eliminate TI-Tregs without perturbing systemic immune homeostasis. Despite the identification of molecules such as CCR8, GPR15, and JMJD1C, which demonstrate relative specificity to TI-Treg, these markers are not exclusively expressed by TI-Tregs. Their presence is also observed in other immune cell subsets or under certain physiological conditions, thereby complicating the development of highly selective therapeutic strategies (Chakraborty and Zappasodi, 2021; Long et al., 2024). Additionally, the complexity of the TME introduces further challenges to achieving specific targeting. Within the TME, complex interactions among various cell types and signaling molecules can substantially affect the distribution and efficacy of targeted therapies (Landry et al., 2018; Fan et al., 2025). Factors such as elevated interstitial pressure, hypoxia (Sun et al., 2025), and the abundance of extracellular matrix (ECM) components may impede the effective delivery of drugs to TI-Tregs, thereby reducing therapeutic efficacy (Zhang et al., 2024). The challenges within the TME extend beyond physical barriers and encompass immune heterogeneity, collectively exerting a significant influence on the efficacy of TI-Treg-targeted therapies. The abundant ECM constitutes a physical barrier within the TME, restricting the penetration of both therapeutic agents and immune cells into the tumor core, thereby limiting therapeutic efficacy. This physical barrier limits the delivery of therapeutic agents to the inner regions of the tumor, consequently constraining overall treatment efficacy. Modulating or degrading the ECM can significantly enhance drug penetration and improve antitumor efficacy (Zhang et al., 2024). Hypoxia represents another critical characteristic of the TME, adversely affecting the efficacy of therapeutic agents and fostering an immunosuppressive microenvironment. It compromises the functional capacity of immune cells and facilitates the accumulation of immunosuppressive cell populations within the TME. The application of oxygen-carrying nanoparticles has been shown to effectively increase local oxygen concentration, thereby mitigating the hypoxic conditions characteristic of the TME and enhancing antitumor efficacy (Sun et al., 2025). In order to develop therapeutic agents that specifically target TI-Tregs, it is essential to thoroughly elucidate the biological distinctions between TI-Tregs and peripheral Tregs, as well as the influence of the TME on these targeted therapies. This understanding will enable the design of more precise and effective treatment strategies (Table 3).

**TABLE 3:** Efficacy and safety of current clinical trials targeting epigenetic regulation of Tregs.

Current progress of the clinical trial	Corresponding article results	Efficacy and safety demonstrated in results
Ongoing recruitment in progress	Epigenetic modulation of Tregs in tumor models	Positive efficacy in improving immune response, minimal adverse effects (Lu et al., 2020)
Ongoing	Combined epigenetic therapy with CAR-T	Enhanced T cell activation and prolonged survival, mild systemic reactions (Wen et al., 2025)
Completed results published	DNMT inhibitors	Prolonged progression-free survival, but hematologic toxicity observed (Liang et al., 2023)
Ongoing	Targeting CCR8 in TI-Treg	Effective reduction in TI-Tregs with minimal systemic effects (García-Díaz et al., 2023)
Ongoing	A2AR antagonists in combination with PD-1 inhibitors	Enhanced tumor control, downregulation of multiple checkpoint molecules on Tregs (Myojin et al., 2023)

## 4 Discussion

Despite significant advances in understanding the epigenetic regulation of Tregs, several critical issues remain under discussion in the study of their epigenetic reprogramming. Current research has demonstrated that epigenetic mechanisms play critical roles in the development, function, and adaptation of Tregs within the TME. However, the causal relationships between specific epigenetic modifications and functional alterations in Tregs are not yet well-defined (Liu K. et al., 2025; Zheng et al., 2025). While DNA methylation of key genes such as *Foxp3, IL2ra,* and *Ilzf2* has been closely linked to Tregs function, the precise molecular mechanisms by which these modifications modulate Treg activity remain inadequately understood. Furthermore, inter-individual and inter-tumoral heterogeneity in these epigenetic patterns necessitates further exploration (Whibley et al., 2019; Ohkura and Sakaguchi, 2020). Additionally, there are divergent perspectives on the interplay between Treg plasticity and epigenetic regulation. Some studies propose that Treg plasticity results from dynamic shifts in epigenetic states, facilitating functional reprogramming in response to varying microenvironmental conditions (Pan and Zheng, 2020). Some scholars contend that cytokine signaling, metabolic states, and other extrinsic factors are the primary determinants of Treg flexibility, with epigenetic regulation serving merely as a secondary or permissive mechanism (Roy et al., 2020; Kang and Zappasodi, 2023). Consequently, the relationship between Treg plasticity and epigenetic modification, as well as the variability of this interaction across different tumor types and individuals, remains an important area for future research. Although several epigenetic drugs have demonstrated therapeutic potential in oncology, their direct effects on TI-Tregs remain largely unexplored. Current research predominantly focuses on the impact of epigenetic drugs on tumor cells themselves, while the broader immune microenvironment-particularly the effects on TI-Tregs and the underlying mechanisms-remains inadequately understood (Pan and Zheng, 2020; Lodewijk et al., 2021; Yang et al., 2023; Liu K. et al., 2025). For instance, HDACi represent a class of epigenetic drugs capable of modulating immune cell function; however, their specific effects on the epigenetic state, survival, proliferation, and functional activity of TI-Tregs within the TME are poorly understood and require systematic investigation (Hull et al., 2016). Furthermore, the diverse effects of various epigenetic drugs on TI-Tregs introduce additional complexity. The underlying mechanisms and signaling pathways necessitate further elucidation to reduce unintended side effects and to formulate personalized combination treatment strategies for patients (Lu et al., 2020; Pan and Zheng, 2020; Wen et al., 2025). In addition to inter-patient variability in drug response, TI-Tregs themselves exhibit differences in suppressive activity depending on individual characteristics and tumor types (Yi et al., 2018; Liu et al., 2020; Gao et al., 2022; Klein et al., 2025). These variations may contribute to heterogeneous responses to epigenetic therapies targeting TI-Tregs. Consequently, future research should focus on identifying appropriate epigenetic drugs and treatment regimens tailored to individual patient profiles to address this challenge. An enhanced understanding of the influence of epigenetic mechanisms on TI-Tregs, along with the identification of common features across different contexts, will be instrumental in optimizing therapeutic strategies for cancer treatment.

The influence of the TME on the epigenetic reprogramming of Tregs is increasingly being elucidated; however, numerous mechanisms and targets remain to be thoroughly investigated (Xu et al., 2011; Macedo-Silva et al., 2019; Field et al., 2020; Renaude et al., 2020; Li Y. et al., 2021; Qu et al., 2023; Yang et al., 2023; Zhu et al., 2023; Long et al., 2024; Mani et al., 2024). The TME represents a highly intricate and dynamic environment, characterized by a multitude of signaling pathways and intercellular interactions that have yet to be fully delineated, each of which may contribute to the epigenetic remodeling of Tregs (Braband et al., 2022; Smiline Girija, 2022). Recent investigations have begun to explore the roles of extracellular vesicles, noncoding RNAs, and the local microbiota in shaping the epigenetic landscape of Tregs; however, the foundational mechanisms and potential for therapeutic intervention remain inadequately understood and warrant further research (Uchida and Bolli, 2018; Russo et al., 2023; Yang et al., 2023; Song et al., 2024). While certain molecules relatively specific to Tregs within the TME have been identified, such as CCR8 and JMJD1C, the development of targeted therapies remains incomplete, and the potential impacts on other physiological functions have not been thoroughly evaluated. Consequently, there is a need for novel targets with enhanced specificity and efficacy, or for more selectively targeted compounds that can overcame tumor heterogeneity while preserving immune homeostasis (Nutt et al., 2007; Bhave et al., 2022; Nie et al., 2024). Targeting the distinct stimulatory signals and transcriptional regulators that are unique to Tregs within the TME presents a promising strategy to precisely modulate TI-Treg function and enhance antitumor immunity. Recent studies suggest that targeting epigenetic regulators such as EZH2, HDAC6, and the lactate signaling pathway could reprogram TI-Tregs, shifting them toward pro-inflammatory phenotype and improving the efficacy of ICIs and CAR-T therapies. In conclusion, a more profound investigation into the mechanisms by which the TME influences the epigenetic landscape of Tregs will facilitate the identification of new therapeutic targets, the development of innovative treatment strategies, and optimizing combination therapies. By targeting multiple epigenetic regulatory pathways, these strategies, particularly those targeting the metabolic and transcriptional reprogramming of Tregs, have the potential to substantially improve cancer immunotherapy approaches. Continued investigation and discovery of these novel mechanisms and targets are anticipated to drive significant advancements and breakthroughs in cancer therapy.
